# Molecular characteristic of *mcr*-1 producing *Escherichia coli* in a Chinese university hospital

**DOI:** 10.1186/s12941-017-0207-z

**Published:** 2017-04-19

**Authors:** Qing-wen He, Xiao-hong Xu, Fang-jun Lan, Zhi-chang Zhao, Zhi-yun Wu, Ying-ping Cao, Bin Li

**Affiliations:** 1 Department of Clinical Laboratory, Fujian Medical University Union Hospital, 29# Xinquan Road, Fuzhou, 350001 Fujian China; 2 The Union Clinical Medical College of Fujian Medical University, Fuzhou, 350004 Fujian China; 3 Department of Pharmacy, Fujian Medical University, Union Hospital, Fuzhou, 350001 Fujian China

**Keywords:** *E. coli*, *mcr*-1, Colistin, Multidrug-resistant

## Abstract

**Background:**

Colistin has been considered as a last-line treatment option in severe infections caused by multidrug-resistant (MDR) gram-negative pathogens. However, the emergence of the mobile colistin resistance gene (*mcr*-1) has challenged this viewpoint. The aim of this study is to explore the prevalence of *mcr*-1 in *Escherichia coli* (*E. coli*) in a Chinese teaching hospital, and investigate their molecular characteristics.

**Methods:**

A total of 700 *E. coli* isolates were used to screen *mcr*-*1* by PCR and sequencing in a Chinese university hospital from August 2014 to August 2015. Susceptibility test of *mcr*-1-producing isolates was determined by Vitek -2 Compact system. 26 virulence factors (VFs), phylogenetic groups, Multi-locus sequence typing (MLST), and DNA Fingerprinting (ERIC-PCR) of strains were investigated by PCR.

**Results:**

Four (0.6%) *mcr*-1 producing *E. coli* isolates were found in this study. The results of antibiotic susceptibility test showed that all four isolates were resistant to colistin, ciprofloxacin, levofloxacin, cefazolin, and trimethoprim/sulfamethoxazole, and were susceptible to amikacin, ertapenem and imipenem. In addition, all 4 isolates exhibited high-level resistance to aztreonam, cefotaxime and gentamicin. The numbers of VFs contained in *mcr*-1 positive isolates were no more than 4 in our study. MLST result demonstrated that these isolates were assigned to two sequence types: ST156 and ST167. The result of phylogenetic analysis showed that four *mcr*-1-positive isolates belong to two phylogenetic groups: A and B1 group. ERIC-PCR showed that four *mcr*-1 positive strains were categorized into three different genotypes.

**Conclusions:**

Our study demonstrated a low prevalence of *mcr*-1 in *E. coli* clinical isolates in a Chinese teaching hospital, and we have gained insights into the molecular characteristics of these *mcr*-1-positive strains. Increasing the surveillance of these infections, as well as taking effective infection control measures are urgently needed to take to control the transmission of *mcr*-1 gene.

## Background

In recent years, colistin has been considered as an effective therapeutic option for the rapid increasing of multidrug-resistant (MDR) gram-negative pathogens [1, 2]. However, the prevalence of the mobile colistin resistance gene (*mcr*-1) in animals and human beings worldwide has challenged this viewpoint [3, 4]. Resistance to polymyxins is mainly caused by the modification to bacterial outer membrane, which was usually considered as chromosomally mediated resistance [5, 6].

Since it was initially found, plasmid-mediated *mcr*-1 has been detected widely [3, 7]. Nowadays, *mcr*-1-producing bacteria have been reported in many regions in China [4, 8]. *Mcr*-1 was firstly found in *Escherichia coli* (*E. coli*), and now it has been spreading to other *Enterobacteriaceae* [9]. Several reports showed that the *mcr*-1 gene could coexist with other resistance genes (such as CRE/ESBL) in *E. coli* and *Klebsiella pneumoniae*, which probably lead to the emergence pan-drug resistant and increase the difficulty of treatment [8, 10]. Therefore, the emergence and spread of *mcr*-1 gene among human beings should be given close attention. The aim of this study was to evaluate the prevalence of *mcr*-1 in *E. coli* clinical isolates in a Chinese teaching hospital, and to investigate the molecular characteristics of these strains.

## Methods

### Bacterial strains

A total of 700 *E. coli* clinical isolates were collected from the clinical laboratory of Fujian Medical University Union Hospital (Fuzhou, Fujian province, China) from August 2014 to August 2015. It is a 2200-bed tertiary care teaching hospital with approximately 95,000 hospital admissions per year, located in southeastern China. All isolates were identified by GNI card of the Vitek system (BioMèrieux, Missouri, France).

### Antibiotic susceptibility testing

Antimicrobial susceptibility testing was performed using the AST-GN16 of Vitek-2 Compact system (Bio Mérieux, France). The antimicrobial agents tested included: tigecycline (glycycline); ertapenem and imipenem (carbapenems); cefazolin; cefoxitin; cefepime, and cefotaxime (cephalosporins); aztreonam (monobactam); amikacin and gentamicin (aminoglycosides); ciprofloxacin and levofloxacin (quinolone); piperacillin/tazobactam; trimethoprim/sulfamethoxazole. The results were interpreted by the Clinical and Laboratory Standards Institute (CLSI) [11]. The MIC of colistin was determined using agar dilution method, and the result was interpreted according to European Committee On Antimicrobial Susceptibility Testing (EUCAST) guidelines [12]. *E. coli* ATCC 25922 was used as a quality control.

### DNA extraction

Several colonies were suspended in 50 µl of sterile distilled water for preparing genomic DNA of the isolates, and then the bacterial suspension was heated at 100 °C for 10 min as described previously [13].

### MCR-1 detection

*mcr*-*1* gene was screened in *E. coli* clinical isolates by PCR using primers as previously described [4]. All of the PCR products were sequenced and then compared with known sequences listed in the GenBank (http://www.ncbi.nlm.nih.gov/blast/).

### Detection of virulence factor genes

Twenty six virulence factors (VFs) genes associated with extraintestinal virulence [14, 15] were detected using a multiplex PCR method as previously described [15]. These genes were as follows: adhesions (*pap*AH, *pap*EF, *pap*C, *pap*G allele I, *pap*G II/III, *pap*G allele II, *sfa/foc*DE, *afa/dra*BC, *fim*H, *gaf*D, *sfa*S, *foc*G and *nfa*E), toxins (*hly*A, *cnf*1 and *cdt*B), siderophores (*fyu*A and *iut*A), protections and invasions (*kps*MTII, *kps*MTIII, *tra*T, *cva*C, kpsMT and K1/K5), miscellaneous (*rfc* and PAI). The PCR products were sequenced and then compared with known sequences listed in the GenBank (http://www.ncbi.nlm.nih.gov/blast/).

### Phylogenetic analysis

The phylogenetic groups (A, B1, B2, and D) of *mcr*-1 producing *E. coli* isolates were identified by a triplex PCR as previously described [16].

### Multi-locus sequence typing (MLST)

*Mcr*-1 positive strains were analyzed by multilocus sequence typing (MLST), which was based on 7 standard housekeeping genes (*adk, fumC, gyrB, icd, mdh, purA, recA*) (http://mlst.ucc.ie/mlst/mlst/dbs/Ecoli) [17].

### DNA fingerprinting

Enterobacterial Repetitive Intergenic Consensus Sequences PCR (ERIC-PCR) was applied to typing *mcr*-1 producing *E. coli* isolates with the primers ERIC-1 and ERIC-2 [18]. DNA fingerprints were compared by visual inspection, ERIC profiles were regarded as different if there were different bands on visual inspection [19].

## Results and discussion

In this study, four isolates (0.6%) were confirmed to carry *mcr*-1 gene, which is lower than previous study [4]. The age of the patients ranged between 38 and 80 years. These *mcr*-1 producing strains were isolated from two different wards (Table 1). Two strains were isolated from the same patient. The clinical data of patients with *mcr*-1 positive *E. coli* infection were shown in Table 2.

**Table 1:** Main characteristics of the *mcr*-1 *E. coli*

Isolates	Data	Ward	Specimen	Phylogenetic groups	MLST	ERIC pattern	VFs	Antibiotic resistance
E321	2014.8	Colorectal surgery	Drainage-fluid	B1	ST156	1	*tra*T, *iut*A	COL, CFZ, FOX, CIP, LVX, SXT, TGC
E684	2015.1	Colorectal surgery	Secretion	B1	ST156	2	*fim*H, *tra*T, *iut*A	COL, CFZ, CTX, FEP, ATM, GEN, CIP, LVX, SXT
E921	2015.4	Hepatobiliary surgery	Secretion	A	ST167	3	*fyu*A, *tra*T, *iut*A	COL, CFZ, CTX, ATM, GEN, CIP, LVX, SXT
E1005	2015.5	Hepatobiliary surgery	Drainage-fluid	A	ST167	3	*fyu*A, *cva*C, *tra*T, *iut*A	COL, CFZ, CTX, ATM, GEN, CIP, LVX, SXT

*CFZ* cefazolin, *FOX* cefoxitin, *CTX* cefotaxime, *FEP* cefepime, *TZP* piperacillin/tazobactam, *ATM* aztreonam, *IPM* imipenem, *ETP* ertapenem, *AMK* amikacin, *GEN* gentamicin, *CIP* ciprofloxacin, *LVX* levofloxacin, *SXT* trimethoprim/sulfamethoxazole, *TIG* tigecycline, *COL* colistin, *MLST* multi-locus sequence typing, *ERIC* enterobacterial repetitive intergenic consensus, *VFs* virulence factor genes

**Table 2:** Clinical data of patients with mcr-1 positive *E. coli* infection

Isolates	Patients	Gender	Age (years)	Underlying diseases	Length of hospital stay (days)	Treatments used	Outcomes
E321	Patient 1	Female	80	Malignancy, hypertension, pulmonary tuberculosis	51	TZP	Survived
E684	Patient 2	Female	57	Perineal infection, hypertension	37	TZP	Survived
E921	Patient 3	Male	38	Hypertension, pancreatitis, diabetes	26	MEM	Survived
E1005	Patient 3	Male	38	Hypertension, pancreatitis, diabetes	26	MEM	Survived

*TZP* piperacillin/tazobactam, *MEM* meropenem

*Mcr*-1 was usually found to be co-localized with other resistance genes on plasmids, such as ESBL genes and carbapenemase genes [20], which might increase the emergence of pan-drug resistance. In our study, the results of antimicrobial susceptibility test showed a high drug resistance in the *mcr*-1-producing isolates. All of the *mcr*-1 positive isolates were resistance to at least 3 different kinds of antibiotics (Table 1).

**Fig. 1:**
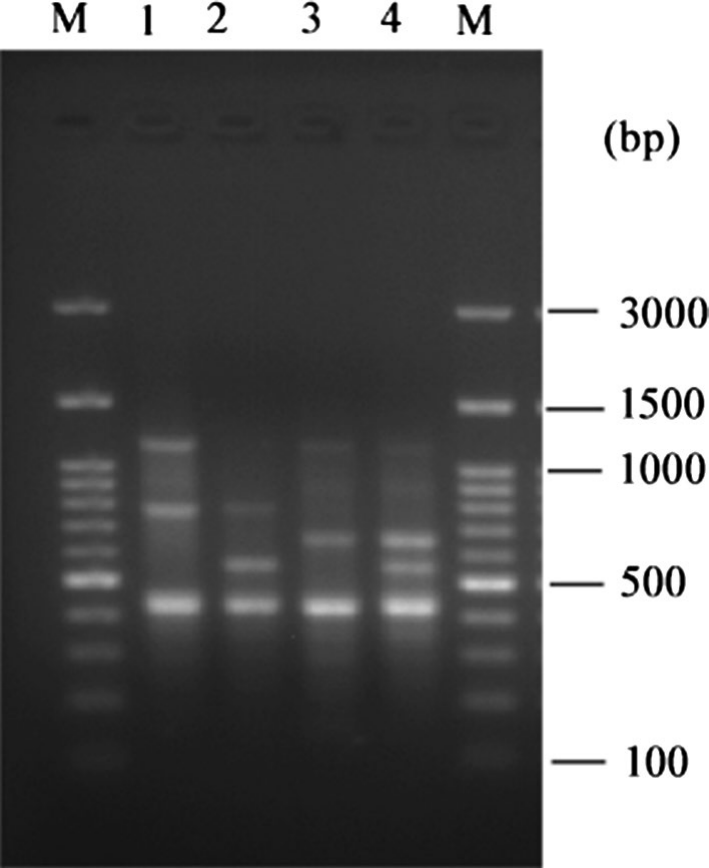
ERIC-PCR products from four mcr-1 positive isolates. *M* mark, *lane 1* E321, *lane 2* E684, *lane 3* E921, *lane 4* E1005

All four *mcr*-1 positive strains detected in our study were resistant to colistin and the MICs ranged from 4 to 16 μg/ml. It will be worrisome once *mcr*-1 coexists with other resistant genes, especially carbapenemase genes because of limited therapeutic options [20]. Previous studies revealed that *mcr*-*1* co-produced with carbapenem-resistant genes in *E. coli* [8, 21]. Fortunately, all of them were susceptible to carbapenems (IPM and ETP), which probably indicated that no carbapenem-resistant genes coexisted with *mcr*-1. Result of ERIC-PCR (Fig. 1) showed that four *mcr*-1 positive strains were categorized into three different genotypes, one of which contained 2 strains (from the same patient). These isolates which have different patterns suggest that they were non-clonal transmission. In a previous study, two *mcr*-1 positive *E. coli* isolates from a single fowl were belonging to phylogenetic B1 and D group [22]. The *mcr*-1-producing isolates in this study were belonged to phylogenetic groups A and B1, which were mainly distributed among human commensal *E. coli* isolates [23]. The *mcr*-1 producing isolates were assigned by MLST to two different sequence types: ST156 and ST167 (Table 1), which was similar to previous reports in other studies from China [8, 22]. *E. coli* ST156 has been found that it has connection with different ESBL genes [24, 25]. ST167 was belonged to ST10 complex and regarded as prevalent ST among ESBL-producing *E. coli* from human and animal sources [26]. In addition, *E. coli* ST167 was reported to be closely related to *bla*_NDM_, which needed closely concern of spreading [27]. The similar molecular characterizations illustrated that *mcr*-1 positive isolates detected from the same department in our study were clonally related.

VFs in *E. coli* were associated with colonization, bacterial fitness and virulence [28]. VFs include five main groups: (1) adhesions; (2) toxins; (3) siderophores; (4) capsule production and (5) protections and invasions. Clinical *E. coli* strains often carry multiple VFs, and isolates belonging to groups A and B1 often have less VFs than those belonging to phylogroups B2 and D [28]. To the best of our knowledge, there is no study concerning about VFs in *mcr*-*1* producing *E. coli*. In our study, *mcr*-*1* producing isolates contained less than 4 different VFs (Table 1). Only five different kinds of VFs had been detected in our *mcr*-*1* positive isolates, which included *fim*H, *fyu*A, *tra*T, *iut*A and *cva*C. *fim*H is one of the most commonly VFs present in *E. coli*, which encodes the adhesion subunit of type 1 fimbriae and related to colonization [15]. Lee et al. reported that *fyu*A, *tra*T, and *iut*A were found to be independent predictors for pathogenicity. Meanwhile, *tra*T and *iut*A were thought to be closely related to ESBL genes [29]. Pitout et al. found that *cva*C was only present in non-CTX-M-producing isolates [30]. Previous reports suggested that antibiotic resistance has negative association with virulence factors [31], which could be interpreted by the loss of VFs associated with mutation to resistance [32].

It is noteworthy that two *mcr*-1 positive *E. coli* strains were isolated from the same patient but at different time (Table 1). Results of MLST and ERIC-PCR revealed that these isolates had identical genetic background. Result of antimicrobial susceptibility test showed that they had similar antibiograms. We speculate that the two isolates probably originated from a same source.

In conclusion, we have revealed a low prevalence of *mcr*-*1* in *E. coli* clinical isolates in a Chinese teaching hospital, and presented detailed molecular characteristics of these isolates. The presence of *mcr*-1 in *E. coli* clinical isolates suggests that it will pose a threat to public healthcare. Effective infection control measures are urgently needed to take to control the transmission of *mcr*-1 gene.

## Abbreviations

MCR-1: mobile colistin resistance gene
MDR: multi-drug resistant
PCR: polymerase chain reaction
CLSI: Clinical and Laboratory Standards Institute
MLST: multi-locus sequence typing
CRE: carbapenem-resistant *Enterobacteriaceae*
ESBL: extended-spectrum β-lactamase
ERIC-PCR: Enterobacterial Repetitive Intergenic Consensus Sequences PCR
VFs: virulence factors
